# Prince of Wales's Hospital Fund for London

**Published:** 1899-12-30

**Authors:** 


### PRINCE OF WALES'S HOSPITAL FUND FOR LONDON.

A meeting of the General Council of the above Fund was held last week at Marlborough House to consider the amounts to be distributed this year, the President (H.R.H. the Prince of Wales) in the chair. Amongst those present were: Lord Rowton, Lord Rothschild, Lord Iveagh, Lord Farquhar, the President of the Royal Society (Lord Lister), the Chairman of the London School Board (Lord Reay), Sir Savile Crossley, Sir Henry Burdett, K.C.B., Cardinal Vaughan, the Chief Rabbi (the Rev. Dr. Adler), the Rev. T. Bowman Stephenson, D.D., Mr. Sydney Buxton, M.P., Mr. Julius Wernher, and Mr. J. G. Craggs.

Sir Savile Crossley, Honorary Secretary, read letters of regret from the following, who were unable to attend: The Bishop of Rochester, Lord Duncannon, the Lord Mayor, Sir Trevor Lawrence, Dr. Church, Mr. H. C. Smith, Mr. John Aird, M.P., Mr. Sandeman, Mr. E. A. Hambro, and the Governor of the Bank of England.

Lord Rothschild, Honorary Treasurer, presented the financial statement, which was as follows:—

Sir,—I have the honour to inform your Royal Highness that the financial position of this Fund up to 19th December is as follows:—

| | | | |
|---|---|---|---|
| Annual subscriptions ... ... ... ... | £25,669 | 0 | 1 |
| Donations and undefined contributions ... | 13,680 | 0 | 2 |
| Annual grant from the trustees of the London Parochial Charities for the Maintenance of Convalescent Hospitals ... ... ... | 1,000 | 0 | 0 |
| Contribution from the League of Mercy ... | 1,000 | 0 | 0 |
| Income from investments ... ... ... | 6,391 | 18 | 5 |
| Sums given by donors to be invested ... ... | 66 | 1 | 2 |
| Subscriptions paid in advance for 1899 ... | 1 | 5 | 0 |
| Making the total receipts ... ... ... | £47,808 | 4 | 10 |
| Deduct expenditure to date ... ... | 1,547 | 11 | 5 |
| Leaves a net amount up to the present of | £46,260 | 13 | 5 |
| The balance of funds from last year is ... | 171,332 | 10 | 3 |
| Making a grand total of ... ... ... | £217,593 | 3 | 8 |

These total funds are held as follows:—

| | | | |
|---|---|---|---|
| Bank of England ... ... ... ... ... | £2,816 | 5 | 11 |
| Other banks and newspaper ... ... ... | 269 | 5 | 0 |
| Investments ... ... ... ... ... ... | 214,400 | 19 | 2 |
| Amount returnable by Inland Revenue in respect of income-tax deducted from income on investments... ... ... ... ... | 100 | 13 | 7 |
| | £217,593 | 3 | 8 |

It is satisfactory to be able to report that about £9,000 more has been received this year.

The expenditure is less, although there has been added this year the cost of a collector and of a large amount of statistical work, including an analysis of the funds held by the whole of the hospitals visited.—I have the honour to remain, your Royal Highness's faithful and obedient servant,

(Signed) Rothschild.

Lord Rothschild: Your Royal Highness will see that the total receipts are £47,808 4s. 10d., from which will have to be deducted the expenditure of £1,547 11s. 5d. I think it is satisfactory to state that the expenditure this year is less than last year, although included in the expenditure is the cost of a collector who was exceedingly useful to us, and who collected a considerable sum of money. I think, considering the numerous calls made on the charity of the nation this year, you have every reason to congratulate yourself on the success of the Fund.

H.R.H. the President: The expenditure having been less, and the income more, I think we can consider it a very satisfactory result.

Lord Rowton, on behalf of Mr. H. C. Smith, read the report of the Executive Council as follows:—

Sir,—The Executive Committee have the honour to inform Your Royal Highness that they have met nine times during the year.

The committee beg to recommend the distribution of £42,000 to 82 institutions. Of this sum £41,000 is allotted to hospitals, £1,000 to convalescent institutions, against £32,500 distributed last year among 59 institutions.

The amount received shows an increase of about £9,000 on the previous year, thus affording proof of the continuous progress of the Fund. It is further only right to state that much of this increase is due to the munificent donations of Mrs. Brydges Willyams and others.

The committee decided that efforts should be made to distribute £50,000 during the current year; it was, however, resolved in October to discontinue further appeals in consequence of the organisation of the war funds.

The expenses of advertising have been still further reduced, and also the costs of the office.

The profits of Sunday concerts were offered to the fund by the Sunday Concert Society and the Crystal Palace Company, but in neither of these instances did any profits result, though the directors of the Crystal Palace Company kindly gave a donation of £100. It has since been decided not to allow the name of the Fund to be used in connection with concerts.

On March 6th, 14th, and 22nd advertisements were inserted in the leading London journals asking all hospitals and convalescent homes desirous of grants to apply within a certain date.

The Convalescent Homes Committee have allotted £1,000 entrusted by the London Parochial Charity Trust for that purpose.

The number of homes applying was 30, the number participating 14. The number of hospitals applying was 87; of these six were ruled out as being outside the limit of seven miles from Charing Cross, or as not being hospitals. It was decided that neither dispensaries nor homes for the dying could be considered, while institutions for incurables could only be considered so far as they dealt with curable cases also.

Eighty-one hospitals were referred to the Visiting Committee, as against 58 last year.

The success of the Fund is largely attributable to the system of visiting, and the thanks of His Royal Highness to the visitors testifies his appreciation of this fact.

Conferences were held with the representatives of four hospitals contemplating rebuilding.

Although 287 beds hitherto closed for the want of means will be now reopened through the action of the Fund and many other improvements carried out, much still remains to be done. There are still nearly 400 beds closed.

The question of the hospital accommodation required in the various districts has not been neglected, and already, as will be seen from this report, the increased requirements of North and South London have been practically dealt with to a partial extent.

The steps likely to be taken to abolish the heavy rating under which the hospitals of the metropolis suffer will doubtless receive the support of the Fund.

The Sunday Fund has already dealt with the yearly profit and loss accounts of the hospitals, and has induced adherence to the uniform system. It is to be hoped that your Royal Highness's Fund will induce every institution to publish a balance-sheet showing the whole of its assets and liabilities.

The Executive Committee desire specially to convey their thanks to the Press and other supporters for their help.

The reports of the Hospitals Distribution Committee and of the Convalescent Homes Committee were received and approved by the executive, and are appended hereto.

I have the honour to be, Sir, your Royal Highness's most obedient, humble servant,

(Signed) H. C. Smith,
Chairman for the Executive Committee.

Sir Savile Crossley read the report of the Hospitals Distribution Committee as follows:—

Sir,—The past year has been one of increased usefulness of the Fund.

The hospitals concerned have been again visited by sub-committees consisting of medical men of wide experience and laymen who take special interest in hospital management. These visitations, while they have admirably answered their immediate object of supplying reliable information regarding the merits and needs of the various institutions, have proved of very high value in stimulating improvements, both in construction and in all departments of administration. Defects have been pointed out, and their remedy has been powerfully promoted by making the grants from the Fund conditional upon the carrying out of the measures that have been recommended.

The suggestions made by the visitors have been almost invariably received in a most friendly spirit, and new interest has been often aroused in those more immediately concerned in the several hospitals, leading them to increase their own subscriptions or to promote in other ways by their individual exertions the needed improvements.

The importance of the services rendered by the Fund has been more generally recognised by the public, so that the sum available for distribution to hospitals this year shows a considerable increase upon last year's amount, the figures being £41,000 now, as compared with £31,500 in the previous twelvemonth.

It is anticipated that the League of Mercy, instituted a year ago by His Royal Highness, will in due time greatly benefit the Fund, but the committee can at present only advise the employment of £1,000 from that source, this being the amount which had been actually paid in when this year's distribution was arranged.

It is recommended that the additional £9,500 now at the disposal of the Fund be employed partly in the way of donations and partly as annual grants which are necessary when money is given for the important purpose of opening closed wards, for which continuous maintenance must be provided.

It has been arranged that 45 beds now unoccupied will be brought into use next year; these added to those thrown open during the last two years give 287 as the number made available to the poor of the metropolis by the agency of the Fund.

The details of the grants and donations recommended for the coming year are given below.

(Signed) Lister.

The awards of the Hospitals Distribution Committee and the report of the Convalescent Homes Committee and their awards were read as follows:—

Grants Recommended by the Distribution Committee.

Alexandra Hospital—Donation, £500. Belgrave Hospital—Donation, £500 (to help the hospital to remove to the South of London). Bolingbroke Hospital—Donation, £1,000 (towards the rebuilding). British Lying-in Hospital—Donation, £100. Cancer Hospital (the committee consider this to be a well-managed hospital, but not in need of a grant this year). Central London Ophthalmic Hospital—Donation, £100 (towards the rebuilding). Central London Throat and Ear Hospital—Donation, £150 (towards an operating-room). Charing Cross Hospital—Annual, £1,000 (in the hope that the improvements required will be put in hand forthwith). Chelsea Hospital for Women—Donation, £250. Cheyne Hospital—Donation, £100 (in consideration of the fact that curable cases are admitted). City of London Chest Hospital—Annual, £1,500 (grant is increased by £500 to enable the reopening of another ward of ten beds). City Orthopædic Hospital—Annual, £250 (the amount named by the authorities as required to open the remaining twenty closed beds). Clapham Maternity—Donation, £250 (towards the building fund). Cottage Hospital, Canning Town—Donation, £100 (towards the building fund). Dental Hospital—Donation, £100. Dreadnought Hospital—Annual, £500; donation, £200 (for the Tropical Diseases Department). East-End Mothers' Home (the income of this institution appears this year sufficient). East London Hospital—Donation, £250. Establishment for Gentlewomen—Donation, £75. Evelina Hospital (no grant is recommended as the income appears sufficient this year). French Hospital—Donation, £100. Gordon Fistula Hospital—Donation, £100 (towards furnishing). Great Northern Central Hospital—Annual, £750 (as before). Grosvenor Hospital—Donation, £100. Guy's Hospital—Annual, £5,000. Hampstead Hospital—Donation, £100 (towards the salary of a house surgeon). Hospital and Home for Incurables, Maida Vale (an institution for incurables only). Hospital for Diseases of the Throat (their claim to be considered another year when their building is completed). Hospital for Epilepsy and Paralysis—Donation, £250 (for rebuilding). Hospital for Sick Children—Annual, £500. Hospital for Women, Soho Square—Donation, £350. Hospital of St. John and St. Elizabeth (no grant is recommended this year. Their case to be considered after the building is opened). Infirmary for Consumption (not considered to be within the scope of this fund as a hospital). Italian Hospital—Donation, £100 (towards furnishing the new building). King's College Hospital—Annual, £1,000; donation, £175 (towards out-patients' department). London Fever Hospital—Donation, £200 (attention to be drawn to the necessity for certain reconstructions). London Homœopathic Hospital—Donation, £200. London Hospital—Annual, £5,000. London Lock Hospital—Donation, £500. London Temperance Hospital—Donation, £500. Memorial Cottage Hospital—Donation, £50. Metropolitan Hospital—Donation, £1,000 (committee trust that the efforts of the authorities will be continued successfully and the debt paid off with the assistance and encouragement shown in this increased grant). Middlesex Hospital—Annual, £1,000 (as before). Mildmay Mission Hospital—Donation, £250. Miller Hospital—Donation, £300. National Hospital for Paralysed—Annual, £750. National Orthopædic Hospital—Donation, £100. New Hospital for Women—Donation, £100 (towards the purposes named by the visitors). North-Eastern Hospital for Children—Annual, £500; donation, £250 (towards building). North London Hospital for Consumption—Annual, £1,000. North-West London Hospital—Donation, £500. Paddington Green Hospital—Donation, £150. Poplar Hospital (an admirably conducted institution, but does not seem to require help this year). Queen Charlotte's Hospital—Donation, £300. Queen's Jubilee Hospital (this institution does not appear to be a hospital in any true sense of the word, and its buildings are quite unsuitable for hospital purposes). Royal Ear Hospital (their claim to be considered when the removal is completed). Royal Eye Hospital—Donation, £100. Royal Free Hospital—Annual, £750; donation, £500 (towards improving the accommodation for nurses). Royal Hospital for Women and Children—Donation, £100 (the committee desire to draw attention to the need for rebuilding). Royal Hospital for Diseases of the Chest, City Road—Donation, £250 (towards improving the nurses' accommodation). Royal London Ophthalmic Hospital—Donation, £750. Royal Orthopædic Hospital—Donation, £250 (towards the expense of rebuilding the hospital on a new site). Royal Westminster Ophthalmic Hospital—No grant this year. Samaritan Free Hospital—Annual, £500. St. George's Hospital (although in view of the financial position of the hospital the committee are unable to recommend a grant, they cannot but express their unqualified approbation of the excellent work that has been done in the way of improving the nursing accommodation and reconstructing the theatre). St. Mark's Hospital (the committee consider that the financial position does not seem to require help this year). St. Mary's Children's Hospital, Plaistow—Donation, £500 (£200 for maintenance, £300 towards the improvements named by the visitors). St. Mary's Hospital, Paddington—Annual, £1,000. St. Monica's Hospital—Donation, £50 (the committee desire to draw attention to the fact that there is no isolation ward). St. Peter's Hospital for Stone—Donation, £50. St. Saviour's Hospital—Donation, £50. St. Thomas's Hospital—Annual, £1,800 (the committee consider that an inquiry officer is needed to prevent abuse by out-patients). Tottenham Hospital—Annual, £1,000 (towards the opening of one closed ward containing about 15 beds); donation, £500. University College Hospital—Annual, £1,400 (as before). Victoria Hospital for Sick Children—Donation, £500 (towards the new building. The committee recommend, in view of the fact that the new building is to cover all the available garden space belonging to the hospital, that a flat roof should be made on the new building for air and exercise). West-end Hospital for Diseases of the Nervous System—Donation, £100 (towards a new operating room). Western Ophthalmic Hospital—Donation, £50. West Ham Hospital—Annual, £300; donation, £250 (towards repairs and alterations necessary). West London Hospital—Donation, £1,000 (the authorities having agreed to carry out the recommendations of the visitors). Westminster Hospital—Annual, £750; donation, £250 (towards the costs of nurses' quarters). Wimbledon Hospital—Donation, £50. Total annual grants, £26,250; total donations, £14,750. (The cheques to be posted on December 29th.)

Sir Henry Burdett: I think, your Royal Highness, the report shows to me (and I have a knowledge of hospitals) the enormous good this Fund is doing. The hon. secretaries are to be congratulated on the accurate way in which they have made their statements, and I believe those statements are calculated to give immense satisfaction to the public, and to immeasurably increase the confidence with which the givers will continue and increase their subscriptions.

H.R.H. the President put to the meeting the adoption of the financial statement and the report of the Executive and Distribution Committees, which was carried unanimously.

H.R.H. the President: I think, gentlemen, all that remains for me to do now is to express my sincere and cordial thanks to the General Council, Visitors, Executive, and honorary officials for the very able manner in which they have carried out their arduous duties. I only hope that next year we may be in a similarly good position. Let us hope that the object we have in view will be carried out so as to enable us to put the hospitals on a sounder basis than hitherto.

Lord Rothschild proposed a vote of thanks to the chairman, which was carried unanimously, and the proceedings then terminated.

